# Decreased serum potassium may disturb sleep homeostasis in essential hypertensives

**DOI:** 10.1038/s41440-018-0131-4

**Published:** 2018-11-16

**Authors:** Mulalibieke Heizhati, Yu Zhang, Liang Shao, Yingchun Wang, Xiaoguang Yao, Suofeiya Abulikemu, Delian Zhang, Guijuan Chang, Ling Zhou, Nanfang Li

**Affiliations:** The Center of Hypertension of People’s Hospital of Xinjiang Uygur Autonomous Region China; The Center of Diagnosis, Treatment and Research of Hypertension in Xinjiang, China. No. 91 Tianchi Road, Tianshan District, Urumqi, Xinjiang, CN 830001 China

**Keywords:** sleep architecture, essential hypertension, serum potassium

## Abstract

The aim is to investigate the association between alterations in the serum potassium (K+) concentration and sleep architecture parameters in essential hypertensives. Two hundred ninety-two hypertensives undergoing polysomnography and providing blood samples were recruited. The sleep architecture was composed of sleep stages 1 (N1), 2 (N2), 3 (N3), 4 (N4) and REM. The light sleep stage (LST) was composed of N1 + N2, and the deep sleep stage (DST) was composed of N3 + N4. The potentialrelationships between electrolytes and sleep parameters were determined via univariate and multivariate analyses. The subjects were divided into two groups via the serum K^+^ median (3.86 mmol/L). The K^+^ < 3.86 mmol/L group showed significantly decreased N1 (7.10 ± 4.55% vs 8.61 ± 5.23%, *p* = 0.002), LST (71.48 ± 11.33% vs 75.92 ± 17.08%, *p* = 0.013), and periodic leg movement during sleep related to microarousals (MA) /arousal (PLMS-A) [4 (1~10) vs 8 (3~15)/night, p < 0.001] and increased REM (17.38 ± 6.43% vs 15.37 ± 6.18%, *p* = 0.007) compared to the K^+^ ≥ 3.86 mmol/L group. A subdivided analysis by gender showed that these changes were more statistically significant in men than in women. Significant positive correlations were identified between K^+^ and N1 (r = 0.169, *p* = 0^.^004), as well as PLMS-A (*r* = 0.222, *p* < 0.001) in subjects. Compared to women, a significantly strong correlation was identified between K+ and REM sleep in men (*r* = 0.158, *p* = 0.028 vs. *r* = 0.078, *p* = 0.442). Multiple linear regression analysis indicated that K+ is significantly associated with N1 in all subjects (*p* = 0.03) and with REM in men (*p* = 0.008), even after adjusting for confounders. Decreased K+ may disturb the homeostasis of the sleep architecture, and gender may interfere with their links in the hypertensive population.

## Introduction

Sleep accounts for approximately one third of the human lifetime, a complex phenomenon that comprises two substrates, non–rapid and rapid eye movement sleep (NREM and REM). NREM is further divided into stages, such as N1, N2, N3 and N4; the latter two stages are also referred to as slow wave sleep [SWS] [1]. The proportion of time an individual spends in each sleep stage represents the sleep architecture.

There has recently been increasing interest in the homeostasis of sleep patterns. Impairment of the sleep architecture is associated with metabolic diseases [2–4], mental diseases [5], and cognitive impairment [6]. The sleep architecture might be influenced by various factors, including external factors, such as environmental factors, and internal factors, such as gender, age, menstrual cycle, exercise [7–11] andphysical diseases. The sleep-wake cycle and sleep architecture, particularly the REM-sleep homeostasis, might also be influenced by neuronal potassium conductance [12, 13]. Accumulating direct and indirect evidence has shown that the activation of potassium channels plays a critical role in sleep regulation [14–16]. In addition, it has been demonstrated that following a reduction of K^+^ levels, the cell surface density of potassium channels leads to accelerated internalization and degradation [17]. However, limited published data have suggested changes in K^+^ levels may influence the sleep architecture and REM sleep [18], although to date, the emollient direct evidence to prove the case is lacking.

In clinical settings, a lower potassium level is common in patients with essential hypertension (EH), resulting from multiple types of pathogenesis, such as abnormal hormone secretion, improper use of diuretics and low K^+^ intake. Previous studies have shown that the incidence of hypokalemia in hypertensives treated with diuretics alone was 10% [19]. Moreover, hypokalemic patients often complain of a poor quality of nocturnal sleep andexcessive daytime sleepiness. Previous data have also shown that patients with abnormal K^+^ levels have more self-reported daytime sleepiness, sleep-related hallucinations and nightmares or abnormal dreams [20]. Potassium supplementation in the diet improves sleep efficiency and sleep fragmentation in healthy individuals [18]. However, it remains unclear whether the sleep-wake cycle and sleep architecture are affected by the concentration of extracellular potassium in EH. Hypertensive patients suffer more sleep disorders than individuals without hypertension. A large population-based study conducted in rural China reported hypertensive subjects had a higher prevalence of poor sleep quality than those without hypertension (36.02% vs 16.29%, respectively) and that the effects of poor sleep quality on hypertension are larger than those of the sleep duration [21].

Therefore, the aim of the present study is to investigate the relationship between serum K^+^ levels and sleep parameters in EH patients.

## Methods

### Subjects

Study subjects were available from 699 hypertensive inpatients (508 men and 191 women) at the Hypertension Center of the People’s Hospital of Xinjiang Uygur Autonomous Region from January to December 2010. The exclusion criteria were as follows: secondary hypertension including primary aldosteronism and renal hypertension, blood pressure ≥ 180/110 mm Hg, congestive heart failure, chronic renal failure, chronic respiratory disease, diabetes mellitus, and a history of psychiatric disorders, systemic disease, thyroid dysfunction, liver cirrhosis, rheumatoid arthritis, sleep diseases, including moderate or severe obstructive sleep apnea [OSA, apnea-hypopnea index (AHI) ≥ 10 events/h], insomnia or shift workers. Only patients who were previously diagnosed as hypertensive and were taking anti-hypertensive medications or were clinically assessed with an auscultatory blood pressure ≥ 140/90 mm Hg for at least 3 times [22] were consideredto have hypertension in the present study.

Two-hundred ninety-two patients were ultimately selected for the present study and were divided into two groups via a median serum K^+^ = 3.86 mmol/L, resulting in the K^+^ < 3.86 mmol/l group (*n* = 145) and the K^+^ ≥ 3.86 mmol/l group (*n* = 147). Weight, height, smoking habits and alcohol use were assessed on the day of polysomnography (PSG). The body mass index (BMI) was calculated as kilograms per meter squared. The data used were approved by the Ethics Committee of the previously cited hospital (Xinjiang, China). All participants provided written informed consent.

### Sleep studies

All patients underwent PSG (Ultrasom, Nicolett, Madison, WI) in a dedicated sleep laboratory, as previously described [23]. The subjects were instructed to go to sleep and wake up according to their usual routines. All data were scored by a qualified sleep technologist licensed by the American Academy of Sleep Medicine. Apnea was defined by the absence of airflow for >10 s. Hypopnea was defined as any airflow reduction that lasted for >10 s and resulted in arousal or oxygen desaturation [24]. Sleep data were manually scored according to the Rechtschaffen and Kales criteria. The following parameters of the sleep architecture were measured: the total sleep time (TST), defined as the time from sleep onset to the end of the final sleep minus wakefulness after sleep onset; the sleep latency, defined as the time from lights out to the first epoch of any sleep; the percentage of each sleep stage out of the TST (stages 1, 2, 3, 4, and REM sleep); the wakefulness after sleep onset (WASO), the time spent awake during the sleep period time (time from sleep onset to the end of final sleep); and the PLMS-A, defined as the event of leg movement that occurred simultaneously or within 3 s followed by MA or awakening [25].

### Echocardiography

Echocardiography was performed using similar methods as previously described [26]. Cardiac ultrasound systems (the iE33 Philips ultrasound system, iU Elite Philips ultrasound system, and iU 22 Philips ultrasound system) were used. The left ventricular (LV) diameters and the interventricular septal wall and posterior wall thickness were measured at end-diastole from M-mode recordings. The ejection fraction and fractional shortening were calculated using standard quantification methods with M-mode measurements from a two-dimensional image. The LV end-diastolic and end-systolic volumes were measured at end-diastole and end-systole from M-mode recordings and were calculated with the Teicholz’s correction of the cube formula.

### Laboratory assessment

Fasting blood samples were collected at 8:00 in the morning. To avoid factitious increases in the measured potassium concentrations, we used a syringe and needle. After venipuncture was achieved, blood was withdrawn in a slow and careful manner for at least 10 s and separated within 30 min [27]. The serum sodium (Na^+^), potassium (K^+^), magnesium (Mg^2+^), calcium (Ca^2+^), chlorine (Cl^-^), total of CO_2_ (TCO_2_), glucose, and creatinine were measured on a C16000 automated biochemistry analyzer (Abbott Laboratories, Abbott Park, IL, USA).

### Statistical analysis

The subjects’ baseline characteristics, electrolytes and sleep parameters were expressed as the mean ± SD. or median (interquartile range). Continuous variables were compared by two-sample t test or Wilcoxon rank sum test, depending on whether the sample had a normal distribution, and the *χ*^2^ test was employed for categorical variables. Correlations between the serum potassium and sleep parameters were calculated with Spearman or Pearson correlations, depending on whether the bivariates were normally distributed. Variables with *p* value < 0.05 were subsequently entered in a multiple liner regression procedure to determine the independent association of K^+^ and sleep parameters [N1, N2, N3, N4 and REM sleep stages]. A *P* value < 0.05 was consideredstatistically significant. All statistical analyses were performed with SPSS statistical software, version 17.0 (Chicago, IL, USA).

## Results

The demographic and polysomnographic characteristics of the subjects in the two groups are shown in Table 1. ubjects in the two groups showed no significant difference in gender composition (men/women: 91/54 vs 102/45, *p* = 0.232), age (45.20 ± 9.88 years vs. 44.93 ± 8.84 years, *p* = 0.807), BMI, Na + , Cl^-^, Ca^2+^, Mg^2+^, total CO_2_, fasting glucose, 24 h urinary creatinine and uric acid, urinary pH, and systolic and diastolic blood pressure. The two groups were also similar in the usage and combination of anti-hypertensive agents, with the exception that the subjects in the K^+^ < 3.86 group took more angiotensin receptor blockers (43.0% vs 29.5%, *p* = 0.019). In addition, the subjects in both groups showed a similar LV function status.

**Table 1:** Baseline characteristics of subjects with K^+^ < 3.86 and K^+^ ≥ 3.86

Characteristics	K^+^ < 3.86 (*n* = 145)	K^+^ ≥ 3.86 (*n* = 147)	Sig. (t / *χ*^2^ / *z*)
Gender (men/women)	91/54	102/45	0.232
Age (years)	45.20 ± 9.88	44.93 ± 8.84	0.807
Body mass index (kg/m^2^)	26.85 (5.26)	27.22 (4.34)	0.506
Systolic blood pressure (mmHg)	136.00 (26)	135.00 (25)	0.357
Diastolic blood pressure (mm Hg)	90.00 (20)	90.00 (16)	0.957
Anti-hypertensive agents			
No medication	45 (31.0)	52 (35.4)	0.431
ACEI (*n*, %)	13 (13.0)	14 (14.7)	0.726
Angiotensin receptor blockers (*n*,%)	30 (30.0)	13 (15.8)	0.019
Calcium channel blockers (*n*, %)	78 (78.0)	72 (75.8)	0.714
Diuretics (*n*, %)	(8.0)	5 (5.1)	0.082
Beta-blockers (*n*, %)	17 (17.0)	13 (13.7)	0.521
Combination of anti-hypertensive agents			
Single (*n*,%)	28 (28.0)	35 (36.8)	0.336
Two (*n*,%)	59 (59.0)	52 (54.7)	
Three (*n*,%)	7 (7.0)	6 (6.3)	
K^+^ (mmol/L)	3.67 (0.31)	4.10 (0.28)	<0.001
Na^+^ (mmol/L)	141.00 (2)	141.00 (3)	0.130
Mg^2+^ (mmol/L)	0.90 (0.10)	0.91 (0.10)	0.244
Ca^2+^ (mmol/L)	2.27 ± 0.12	2.26 ± 0.11	0.431
CI^−^ (mmol/L)	104.00 (3.00)	105.00 (3.00)	0.086
HCO_3_− (*n* = 20)	23.05 (3)	21.65 (2)	0.165
Total CO_2_ (mmol/L)	23.60 (3)	23.90 (4)	0.847
Fasting glucose (mmol/L)	4.86 (1.00)	4.80 (1.00)	0.178
24 h Creatinine (umol/L)	70.00 (24.00)	68.00 (23.00)	0.094
24 h Uric acid (umol/L)	345.00 (140.00)	337.50 (115.00)	0.268
Urinary pH	6.00 (1)	5.50 (1)	0.148
Left ventricular systolic geometric and systolic function parameters			
LV end-diastolic diameter (mm)	46.00 (6.00)	47.00 (5.00)	0.068
LV end-systolic diameter (mm)	24.00 (3.00)	25.00 (3.00)	0.183
LV end-diastolic volume (ml)	67.00 (24.00)	70.00 (20.00)	0.230
LV end-systolic volume (ml)	42.00 (14.00)	43.00 (13.00)	0.186
LV ejection fraction (%)	60.00 (6.00)	60.00 (7.00)	0.314
Interventricular septum (mm)	10.00 (1.00)	10.00 (1.00)	0.961
LV posterior wall (mm)	10.00 (1.00)	10.00 (1.00)	0.887

*ACEI* Angiotensin-converting enzyme inhibitors

As shown in Table 2, the K^+^ < 3.86 mmol/l group showed significantly lower N1 (7.10 ± 4.55 vs 8.61 ± 5.23%, *p* = 0.002), LST (71.48 ± 11.33 vs. 75.92 ± 17.08%, *p* = 0.013), PLMS-A [4 (1–10) vs. 8 (3–15), *p* < 0.001] and significantly higher REM (17.38 ± 6.43 vs 15.37 ± 6.18%, *p* = 0.007) than the K^+^ ≥ 3.86 mmol/L group. No statistically significant differences were identified in the AHI, TST, sleep efficiency, sleep latency, WASO, N2, N3, N4, and DST between the groups.

**Table 2:** Clinical characteristics for the sleep studies in subjects with K^+^ < 3.86 and K^+^ ≥ 3.86

Characteristics	K^+^ < 3.86 (*n* = 145)	K^+^ ≥ 3.86 (*n* = 147)	Sig. (*t* / *χ*^2^ / *z*)
Total sleep time (min)	372.53 ± 83.66	384.97 ± 74.37	0.180
Sleep latency (min)	41.49 ± 45.77	47.84 ± 50.05	0.148
Wake time after sleep onset (min)^a^	4.12 ± 0.82	4.14 ± 0.78	0.870
N1 (%)	7.10 ± 4.55	8.61 ± 5.23	0.002
N2 (%)	64.38 ± 9.86	66.19 ± 8.66	0.092
N3 (%)	3.1 (5)	2.8 (4)	0.688
N4 (%)	5.9 (11)	4.9 (10)	0.282
LST (%)	71.48 ± 11.33	75.92 ± 17.08	0.013
Deep sleep stage (%)	9.3 (14.5)	8.4 (14.4)	0.419
Rapid eye movement (%)	17.38 ± 6.43	15.37 ± 6.18	0.007
Apnea hypopnea index (events/h)	3.9 (6)	3.6 (5)	0.157
Lowest saturation of oxygen (%)	83.81 ± 8.17	85.01 ± 4.76	0.988
PLMS-A(events/night)	4 (9)	8 (12)	< 0.001
Sleep efficiency (%)	74.81 ± 9.66	75.49 ± 8.48	0.527

*PLMS-A* periodic leg movement during sleep related to microarousals (MA)/arousal, events/night

^a^Log transform

**Fig. 1:**
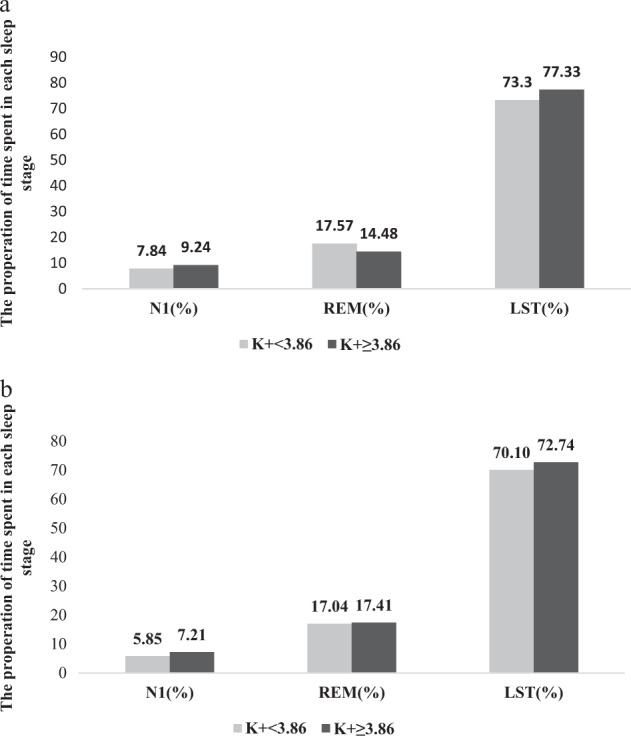
**a**. Sleep stages between different serum potassium levels in male subjects. **b**. Sleep stages between different serum potassium levels in female subjects

As shown in Fig. 1a, b, when the subjects were subdivided by gender, the hypertensive men with K^+^ < 3.86 mmol/l showed significant decreases in the N1, LST, and PLMS-A and a significant increase in REM sleep compared to the women.

**Fig. 2:**
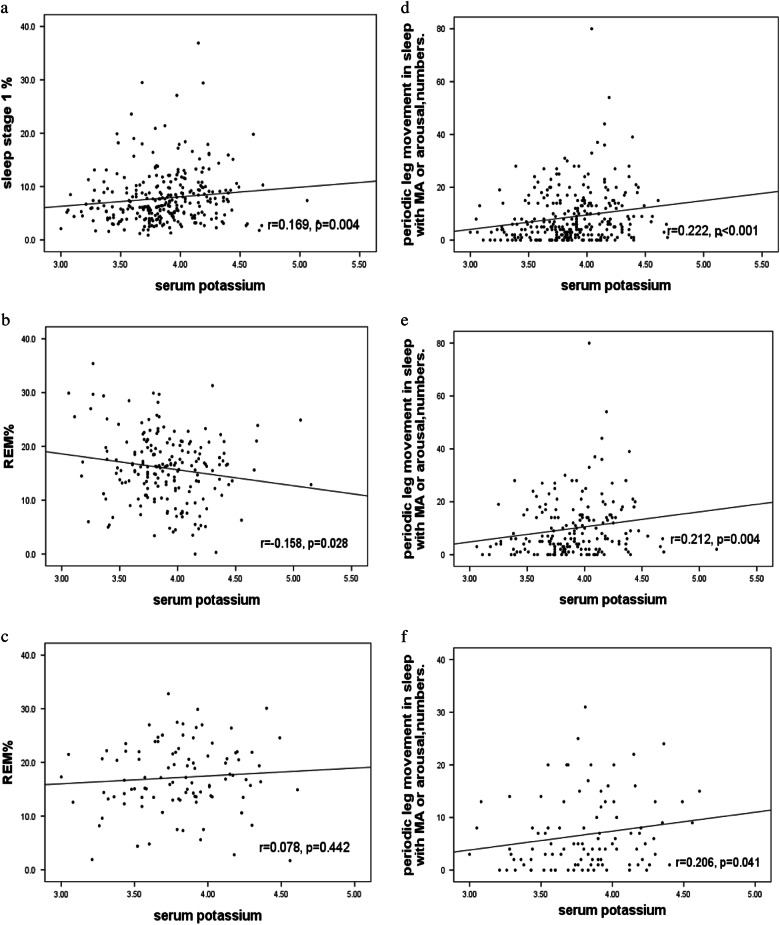
**a** Correlation between sleep stage 1% and serum potassium in all subjects. **b** Correlation between REM% and serum potassium in male subjects. **c** Correlation between REM% and serum potassium in female subjects. **d** Correlation between PLMS-A and serum potassium in all subjects. **e** Correlation between PLMS-A and serum potassium in male subjects. **f**. Correlation between PLMS-A and serum potassium in female subjects

Significantly positive correlations were identified between K^+^ and N1 (*r* = 0.169, *p* = 0.004) (Fig. 2a) and PLMS-A (*r* = 0.222, *p* < 0.001) (Fig. 2d) in all patients. When subdivided by gender, significant correlations were identified between K^+^ and PLMS-A in men (r = 0.212, *p* = 0.004) (Fig. 2e) and women (*r* = 0.206, *p* = 0.041) (Fig. 2f). Moreover, a significant negative correlation was identified between K^+^ and REM sleep (*r* = -0.158, *p* = 0.028) (Fig. 2b) in men, rather than in women (*r* = 0.078, *p* = 0.442) (Fig. 2c).

As shown in Table 3, multiple linear regression analysis indicated that K^+^ is associated with N1 in all subjects (*p* = 0.03) and with REM in men (*p* = 0.008), even after adjusting for age, BMI, AHI, SBP, DBP, Na^+^, Cl^-^, Ca^2+^, Mg^2+^ and fasting glucose with/without smoking history as potential confounders.

**Table 3:** The multiple linear regression between sleep parameters and K^+^

	dependent variable	K^+^	95%CI		*P* value
		Unstandardized Coefficients B	lower	upper	
Model 1	N1 (total)	1.936	1.049	2.823	0.030
Model 2	N1 (male)	1.567	0.383	2.751	0.187
Model 3	N1 (female)	1.838	0.644	3.032	0.127
Model 4	REM (total)	−1.820	−2.989	−0.651	0.121
Model 5	REM (male)	−3.822	−5.252	−2.392	0.008
Model 6	REM (female)	3.110	1.005	5.215	0.143

Model 1–3: we used N1 as dependent variable, multiple liner regression model with indicator variables for age, BMI, AHI, SBP, DBP, K^+^, Na^+^,Cl^-^, Ca^2+^, Mg^2+^, TCO_2_ and fasting glucose as independent variables in patients respectively(stepwise methods). Model 4–6: we used REM as dependent variable, multiple liner regression model with indicator variables for age, BMI, smoking history, AHI, SBP, DBP, K^+^, Na^+^,Cl^-^, Ca^2+^, Mg^2+^, TCO_2_ and fasting glucose as independent variables in patients respectively(stepwise methods).

## Discussion

To our knowledge, this investigation is the first study to propose circulating K + concentrations may disturb the sleep pattern assessed by PSG in essential hypertension. The primary findings are as follows: (1) compared to EH subjects with higher serum K^+^, the N1% was shortened and K^+^ had a significantly positive independent association with N1% in EH subjects with lower serum K^+^. (2) Compared to EH men with higher serum K^+^, REM sleep was prolonged and the serum K^+^ had a significantly negative independent association with REM sleep in those with lower serum K^+^.

It was seemingly difficult to wake up individuals with a lower potassium from sleep onset in our population. N1 sleep is a transition from wakefulness or nocturnal arousal to sleep onset. Normally, when arousal occurs, N2, N3, N4 or REM sleep is likely to change to N1 sleep according to the Rechtschaffen and Kales scoring rules [28]. Therefore, decreased N1 sleep possibly implicates the reduction of cortical activity or movement arousal and WASO. Our findings show that although the natural logarithm of WASO is not significantly different, the PLMS-A is significantly lower in the K^+^ **<** 3.86 mmol/l group than inthose with higher serum potassium. It is established that the extracellular K^+^ concentration plays a pivotal role in mediating the movement of potassium in and out of skeletal muscle. Therefore, patients with hypokalemia coincide with lower excitability and contractility of the skeletal muscles. Accordingly, we speculate that it is not easy to wake up individuals with lower serum K^+^ because of a possibly high arousal threshold, and the decrease in the PLMS-A may be the crux of the problem. Furthermore, potassium channels play an important role in regulating the activity of neuronal pathways by influencing the resting membrane potential of neurons. The activity of potassium channels has been found to both regulate neurotransmitter release and mediate the effects of neurotransmitter activation [29]. Therefore, we speculate that a decrease in N1 sleep associated with lower serum K^+^ may possibly be due to a decrease in neurotransmitter release and the conduction of neuronal pathways.

The REM sleep time might be lengthened pathologically in the K^+^ < 3.86 mmol/l group. In clinical settings, essential hypertensive patients often present with hypokalemia. The prevalence of hypokalemia (K^+^ < 3.5 mmol/l) in 292 essential hypertensive patients is 13.7%, which is less than Cohn’s report ( > 20% in hospitalized patients) [30]. Potassium channels have also been found to contribute to the thalamocortical neuron activity involved in regulating the sleep stage and cognition [31]. Pang et al showed that both REM-associated theta oscillations and the REM sleep time are suppressed in K^+^ channel-mutated mice [32]. The K^+^ level may influence the activity of potassium channels; therefore, we speculate that the activity or density of potassium channels might be decreased and the K^+^ conductance may also be reduced with declined K^+^, which, in turn, may also lead to a reduction in REM sleep, followed by compensatory REM rebounds. Consequently, the total REM sleep time may be pathologically lengthened.

The clinical implication of our findings is that compared with NREM sleep, REM sleep is associated with greater sympathetic activity and cardiovascular instability in healthy subjects and EH patients [33, 34]. Although REM is important for memory formation [35, 36] and to process emotional information [37, 38] and it is necessary for brain development, if excessively lengthened, it may produce a series of problems. For example: 1) nocturnal BP may be increased, resulting in an abnormal BP circadian rhythm; 2) hormones associated with sympathetic nervous excitement may be increased; and 3) nightmares may be related to an altered sleep architecture, particularly lengthened REM sleep [39], which may explain, in part, why these types of subjects have more sleep-related hallucinations and nightmares or abnormal dreams.

Gender is the most important interfering factor for the sleep structure, the mechanisms of which remain unclear. Consistent with previous data [40], our findings suggest significant differences in the sleep architecture of both genders potentially due to sex hormones affecting sleep regulatory mechanisms. Sleep disorders, such as OSA and insomnia, exhibit significant gender disparities [41, 42]. While hypokalemia is more likely to affect the sleep architecture of EH men, the causal pathway of the two is unclear. Further studies are required to investigate the underlying mechanisms that link serum potassium, gender and sleep architecture in EH.

While explaining the current results, the effects of anti-hypertensive agents and renal and heart function on serum kalemia should be considered. It is reported that >20% of hospitalized patients have hypokalemia (<3.5 mmol/L) [30]. However, in the current study, the rate is 13%, and the types and combinations of anti-hypertensive agents in the current population were similar, with the exception that the subjects in the lower potassium group were taking more ARBs. Moreover, it is also a reflection of the clinical setting, where physicians are more willing to prescribe ACEI and ARBs to hypertensives with lower potassium [43]. In addition, the indicators of left ventricular function and renal function were not significantly different between the groups; therefore, it may be reasonable to assume the extracellular volume levels were similar between the groups and exclude their potential effects on the current results.

There are several limitations in this study. First, the nature of the cross-sectional study does not enable conclusions to be drawn. Second, the sleep architecture is measured during a single night PSG, which may not be representative of the habitual sleep pattern of subjects. Taking full account of this problem, study subjects underwent PSG in a dedicated sleep laboratory with suitable temperature and humidity, and patients with ≥65% of the sleep efficiency and ≥300 min of TST were included in the study. Another potential limitation of our study is that the weak link established between K^+^ and sleep parameters may be due to the lack of healthy controls.

## Conclusion

Our findings provide evidence that hypokalemia may disturb the sleep architecture in hypertensive individuals, particularly in men with EH.
